# Impact of Vanadium–Titanium–Magnetite Mining Activities on Endophytic Bacterial Communities and Functions in the Root Systems of Local Plants

**DOI:** 10.3390/genes15050526

**Published:** 2024-04-23

**Authors:** Zhuang Xiong, Yunfeng Zhang, Xiaodie Chen, Ajia Sha, Wenqi Xiao, Yingyong Luo, Lianxin Peng, Liang Zou, Qiang Li

**Affiliations:** Key Laboratory of Coarse Cereal Processing, Ministry of Agriculture and Rural Affairs, Sichuan Engineering & Technology Research Center of Coarse Cereal Industrialization, School of Food and Biological Engineering, Chengdu University, Chengdu 610106, China; xiongzhuang2000@126.com (Z.X.); zhangyunfeng@cdu.edu.cn (Y.Z.); cxd0512@126.com (X.C.); shaajia19980108@126.com (A.S.); xwq990713@126.com (W.X.); lyy1478963@126.com (Y.L.); penglianxin@cdu.edu.cn (L.P.); zouliang@cdu.edu.cn (L.Z.)

**Keywords:** mining activity, soil pollution, bioremediation, microecology

## Abstract

This study utilized 16S rRNA high-throughput sequencing technology to analyze the community structure and function of endophytic bacteria within the roots of three plant species in the vanadium–titanium–magnetite (VTM) mining area. The findings indicated that mining activities of VTM led to a notable decrease in both the biodiversity and abundance of endophytic bacteria within the root systems of *Eleusine indica* and *Carex* (*p* < 0.05). Significant reductions were observed in the populations of *Nocardioides*, concurrently with substantial increments in the populations of *Pseudomonas* (*p* < 0.05), indicating that *Pseudomonas* has a strong adaptability to this environmental stress. In addition, β diversity analysis revealed divergence in the endophytic bacterial communities within the roots of *E. indica* and *Carex* from the VTM mining area, which had diverged to adapt to the environmental stress caused by mining activity. Functional enrichment analysis revealed that VTM mining led to an increase in polymyxin resistance, nicotinate degradation I, and glucose degradation (oxidative) (*p* < 0.05). Interestingly, we found that VTM mining did not notably alter the endophytic bacterial communities or functions in the root systems of *Dodonaea viscosa*, indicating that this plant can adapt well to environmental stress. This study represents the primary investigation into the influence of VTM mining activities on endophytic bacterial communities and the functions of nearby plant roots, providing further insight into the impact of VTM mining activities on the ecological environment.

## 1. Introduction

Vanadium–titanium–magnetite (VTM) is a common polymetallic ore that includes a range of metals, including iron (Fe), vanadium (V), titanium (Ti), cobalt (Co), and chromium (Cr), with Fe, V, and Ti being the predominant elements present in VTM [1]. VTM is a significant source of Fe, as well as a primary source of V and Ti. Therefore, it has significant value for comprehensive utilization [2]. The world’s VTM resources are primarily found in China, Canada, Australia, Russia, New Zealand, and South Africa, totaling a global reserve of almost 48 billion tons [3,4,5,6]. China has reserves of 9.38 billion tons of VTM resources, primarily located in the Panxi region, with the most substantial reserves of 3.6 billion tons found in the Hongge reserves in the Panzhihua–Xichang region [7,8,9,10]. V and Ti mainly exist in VTM as associated ores. V and Ti are crucial rare metals that find extensive applications in steel, metallurgy, chemicals, batteries, aerospace, and various other industries [11,12,13,14]. In the iron and steel industry, V is a commonly utilized component. The addition of V to steel can improve the wear resistance, strength, hardness, ductility, and other properties of steel [11]. Ti and its alloys have excellent properties, such as corrosion resistance, high strength, good strength at high and low temperatures, non-magnetic properties, good adaptability to humans, shape memory, and superconductivity [15]. Due to its lightweight and high-strength characteristics, it has been widely used in aerospace and other fields. In recent years, its application has gradually expanded to include shipbuilding, petrochemical equipment, offshore platforms, electric power equipment, medical treatment, and high-end consumer goods [15,16]. With the increasing demand for V and Ti, the mining scale of VTM is also increasing. Nevertheless, during mineral mining and ore processing processes, significant quantities of VTM tailings are produced, which are frequently disposed of in dams, resulting in various environmental consequences, including the discharge of harmful contaminants into the nearby surroundings. These conditions pose a danger to the local ecosystems [17,18,19]. However, the impact of VTM mining on local plants and microorganisms has not been determined.

Endophytic bacteria, which are present in healthy plant tissues, are essential microbial-plant symbionts and valuable microbial resources that do not pose a significant threat to the host [20,21,22,23]. Multiple studies have proven that a high quantity of endophytic bacteria can live within plant tissues, developing a variety of mutually beneficial symbiotic partnerships [24,25,26]. While colonizing plant tissues, these microbes may also enhance plant adaptability in contaminated soil [27,28]. Moreover, endophytic bacteria can promote seed germination and growth through various mechanisms [29,30,31,32,33,34]. Some *Bacillus*, *Pseudomonas*, and *Pantoea* species can synthesize secondary metabolites with antibacterial properties, exhibiting strong antagonistic effects against plant pathogenic bacteria [35,36,37]. Furthermore, endophytic bacteria are essential components of plant microbial communities and possess substantial potential for use in protecting plants [38]. Endophytic bacteria, like beneficial microbes present in different ecosystems, can function as transporters for emerging bioactive substances, thereby holding importance in medicine, agriculture, and industry [22,25,39]. The diversity of plant endophytes and the establishment of community structures are closely related to factors such as plant variety, growth environment, and geographic location [40]. Endophytic bacteria, which are associated with plant health and productivity, have become a focal point of interest in scientific and commercial realms worldwide in recent years [22,41,42]. However, the effects of VTM mining on the diversity and function of endophytic bacteria in the root systems of nearby plants, as well as the response strategies of root endophytes, have not been determined.

In this study, three local plant species (*Dodonaea viscosa*, *Carex* L., and *Eleusine indica*) were used as the research objects, and the influence of these three plant species on the diversity and function of the endophytic bacterial communities in the roots was investigated through 16S rRNA high-throughput sequencing. Meanwhile, the response strategies of these three plant root endophytic bacteria to VTM mining were analyzed. This study addresses the gap in understanding the ecological effects of VTM mining and provides insights into the ecological remediation of pollution caused by VTM mining.

## 2. Materials and Methods

### 2.1. Collection of Plant Roots and Extraction of Endophytic Bacterial DNA from the Roots

The sampling site was located in the VTM mining area near Hongge Village (101°56′ E, 26°32′ N), Yanbian County, Panzhihua City, Sichuan Province, China. The site has an average annual rainfall of 1065.6 mm, an average annual temperature of 19.2 °C, an average annual sunshine of 2307.2 h, and a relative humidity of 66.6%. We chose three different species of plants with the best local growing conditions for our study: *D. viscosa*, *Carex*, and *E. indica*. To collect the plants, we used the five-point sampling method. First, the midpoint of the diagonal line was determined as the center sampling point. Next, four points were selected on the diagonal line with a distance of 20 m from the center sampling point as sample points. Three plants of each species were randomly collected and a total of 45 plants were collected. In addition, we used the same sampling method to collect these three plants as control samples from the soil of a non-VTM mining site located 3 km away from the mine site. Extracted from the soil, the roots of these three plants had the adhering soil shaken off to obtain the root systems. The plants were subsequently placed in sterile sample bags filled with ice packs, shipped to the laboratory, refrigerated at 4 °C, and treated within 24 h. The residual soil on the surface of the plant roots was washed with sterile distilled water, soaked in 70% ethanol for surface disinfection, and finally rinsed repeatedly with sterile distilled water. All plants were treated in this manner, and each plant had three biological replicates. About 50× *g* of plant roots were weighed from each sample into a 15 mL sterile centrifuge tube for bacterial diversity analysis. Following this, 18 samples were transferred to the laboratory in a foam box with ice packs for DNA extraction and 16S rRNA sequencing. Genomic DNA was extracted from plant root samples by utilizing the Omega Bio-Tek Plant DNA Kit (Norcross, GA, USA). The DNA was then separated on a 1% (*w*/*v*) agarose gel for the purpose of evaluating the quality of the extracted DNA. The samples of *D. viscosa*, *Carex*, and *E. indica* from the VTM mining area were represented as VT-Dvi, VT-Car, and VT-Ein, respectively. The control samples of these three plant species were represented as CK-Dvi, CK-Car, and CK-Ein, respectively.

### 2.2. PCR Amplification and Detection

The concentration of DNA was quantified by Nanodrop One. The extracted genomic DNA was diluted to 1 ng/µL with sterile water, and the 16S rRNA V3-V4 region of the sample was subsequently carried out with primers that included a barcode (341F: 5′-cctacgggaggcagcagg-3′; 806R: 5′-GGACTACNVGGGTWTCTAAT-3′). PCR was performed using 15 μL of Phusion^®^ High-Fidelity PCR Master Mix from New England Biolabs, 2 μL of forward and reverse primers, and 10 ng of template DNA. During thermal cycling, denaturation at 98 °C for 1 min was repeated 30 times, including denaturation for 10 s, annealing at 50 °C for 30 s, extension at 72 °C for 30 s, and storage at 72 °C for 5 min. After completion of PCR, the products were first mixed with an equal volume of loading buffer containing SYBR Green, after which the PCR products were detected via electrophoresis on a 2% (*w*/*v*) agarose gel. When amplification was complete, the PCR products were purified using the Qiagen Gel Extraction Kit (Qiagen, Hilden, Germany).

### 2.3. Library Preparation, Sequencing, and Raw Data Processing

As per the manufacturer’s guidelines, we proceeded to generate sequencing libraries using the TruSeq^®^ DNA PCR-Free Sample Preparation Kit (Illumina, San Diego, CA, USA), which included index codes. For assessing the library’s quality, we used both a Qubit@2.0 Fluorometer (Thermo Scientific, Waltham, MA USA) and an Agilent Bioanalyzer 2100 system. Subsequently, the library underwent sequencing with the Illumina NovaSeq platform to yield a 250 bp paired-end sequence. The reads were identified according to their individual barcodes. Following this, the barcodes and primer sequences were eliminated. The merging of paired-end reads was carried out using FLASH V1.2.7 [43]. To obtain high-quality labels, the original labels underwent processing following the quality control procedure of QIIME V1.9.1 [44]. After comparing the labels to the Silva reference database, the dataset was cleansed of the identified chimeric sequences [45].

### 2.4. OTU Cluster and Species Annotation

By utilizing Uparse v7.0.1001, sequences exhibiting a similarity of 97% or higher were categorized into operational taxonomic units (OTUs) [46]. For the purpose of annotation facilitation, a specific sequence was chosen to represent each OTU. The representative sequences were assigned the classification information from the Silva database utilizing the Mothur algorithm [45]. To analyze the phylogenetic relatedness of OTUs and the variation in dominant species among different samples or taxa, we utilized MUSCLE v3.8.3 for multiple sequence alignment [47]. Normalization of OTU abundance data was carried out by aligning it with a benchmark sample that had the fewest sequences. Utilizing the normalized data, further analyses were conducted on α and β diversity.

### 2.5. α and β Diversity Analyses

We used seven indicators, Pielou evenness, Simpson, Chao1, observed species, Shannon, Good’s coverage, and Faith_pd, to analyze species diversity in each sample. For the calculation of these indicators, QIIME 1.7.0 software was used, and the results were displayed using R v2.15.3 [44]. For community richness evaluation, we employed three indicators: Pielou evenness, Chao1, and observed species. For community diversity assessment, we utilized the Shannon and Simpson indices. β diversity analysis was employed to examine the changes in species complexity among the samples. Lastly, the R vegan software was used to conduct Non-metric Multidimensional Scaling (NMDS) analysis and Principal Coordinates Analysis (PCoA).

### 2.6. Functional Prediction of Endophytic Bacteria

PICRUSt2 can elucidate the functions of endophytic bacteria within the root system [48,49], which utilizes the Gene Ontology (GO), KO, and MetaCyc databases [50,51]. Before the cluster analysis, we employed the FactoMineR and ggplot2 software packages in R v2.15.3 to conduct PCoA in order to decrease the dimensionality of the original variables.

### 2.7. Statistical Analysis

Statistical analysis was employed to identify the significance of differences among the samples. The *t*-test was applied for the direct comparison of two groups of samples, while Tukey’s test was used to analyze multiple sample groups. A *p*-value below 0.05 signifies a statistically significant difference between the groups.

## 3. Results

### 3.1. High-Throughput Sequencing Statistics of Endophytic Bacterial Communities

**Figure A1:**
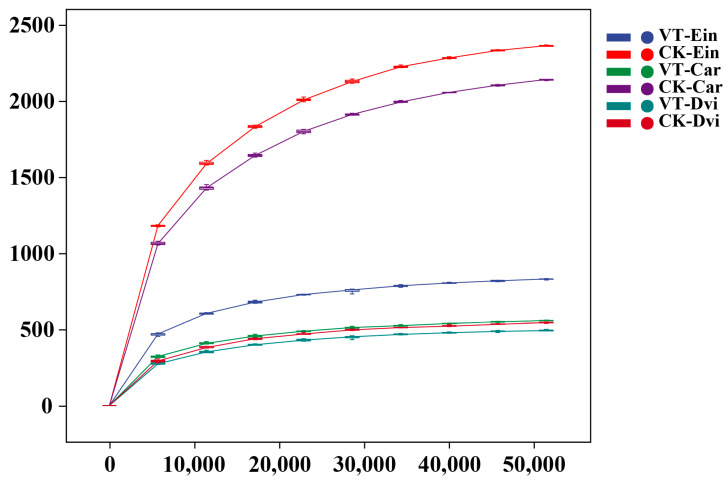
Rarefaction curves of root endophytic bacteria OTUs in different samples. VT-Dvi, *D. viscosa* in the VTM mining area; VT-Car, *Carex* in the VTM mining area; VT-Ein, *E. indica* in the VTM mining area; CK-Dvi, *D. viscosa* in the non-VTM mining area; CK-Car, *Carex* in the non-VTM mining area; CK-Ein, *Eleusine indica* in the non-VTM mining area.

Utilizing 16S rRNA high-throughput sequencing technology, we examined the endophytic bacteria within the root systems of *D. viscosa*, *Carex*, and *E. indica* samples obtained from both the VTM mining region and the non-VTM mining region in this study. In Figure A1, the curve for the sparse OTUs is displayed. The incremental increase in the observed species was positively associated with the growing quantity of sequencing reads. When the number of sequencing reads reached over 20,000, the sparse curve gradually stabilized, indicating that the sequencing results were close to saturation. There were enough reads to effectively capture the comprehensive profile of endophytic bacteria residing in the root system of the sample. Therefore, the large amount of sequencing data can provide insight into the bacterial diversity present in the sample. After removing chimeras, poor-quality reads, and short reads, an average of 97,932 clean reads were successfully acquired per sample. These clean reads were then allocated to OTUs using a 97% similarity threshold. The number of OTUs per sample varied across all samples, ranging from a minimum of 380 to a maximum of 1283, with the average number across all samples being 723 OTUs.

### 3.2. α Diversity Indices

**Figure 1:**
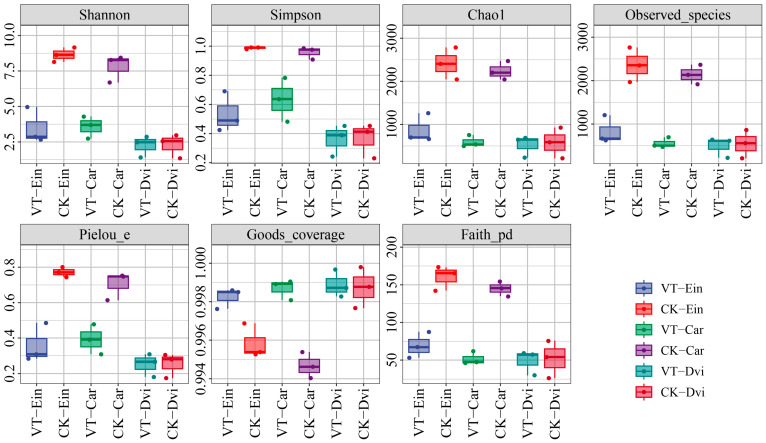
Box diagram of the differences in the α diversity indices of endogenous root bacteria between different samples. Colored dots represent values that are significantly different from other data in this group. VT-Dvi, *Dodonaea viscosa* in VTM mining area; VT-Car, *Carex* in VTM mining area; VT-Ein, *Eleusine indica* in VTM mining area; CK-Dvi, *Dodonaea viscosa* in non-VTM mining area; CK-Car, *Carex* in non-VTM mining area; CK-Ein, *Eleusine indica* in non-VTM mining area.

The diversity and richness of the samples were evaluated using seven indicators (Shannon, Pielou evenness, Simpson, Chao1, observed species, Good’s coverage, and Faith_pd), as illustrated in Figure 1. A higher α diversity index indicates a greater level of community diversity among the endophytic bacteria present in the root system of the sample. The Shannon and Simpson indices showed that the CK-Ein and CK-Car samples had the highest levels of community diversity, while the VT-Dvi and CK-Dvi samples had the lowest levels of community diversity. The VTM mining activities significantly reduced the Shannon and Simpson indices of the VT-Ein and VT-Car samples compared to those of the CK-Ein and CK-Car samples (*p* < 0.05). In contrast to the CK-Dvi sample, the mining activities conducted by VTM did not have a notable impact on the community diversity of the VT-Dvi sample. In addition, an analysis of community richness, including observed species, Chao1, and Pielou evenness, showed that the CK-Ein sample exhibited the greatest diversity, while the VT-Car sample showed the least. The Chao1, observed species, and Pielou evenness indices of endophytic bacteria in the root system of *E. indica* and *Carex* within the VTM mining area exhibited a significant decrease compared to those in the non-VTM mining area (*p* < 0.05). However, VTM mining did not significantly impact the diversity of endophytic bacteria within the root system of *D. viscosa*. In comparison to the CK-Ein and CK-Car samples, the Faith_pd index was significantly lower in the VT-Ein and VT-Car samples (*p* < 0.05), while the Good’s coverage index was notably higher (*p* < 0.05). Nevertheless, when compared to the samples of VT-Dvi and CK-Dvi, there were no significant changes in any of the indices.

### 3.3. Taxonomic Analyses of the Endophytic Bacterial Communities

**Figure 2:**
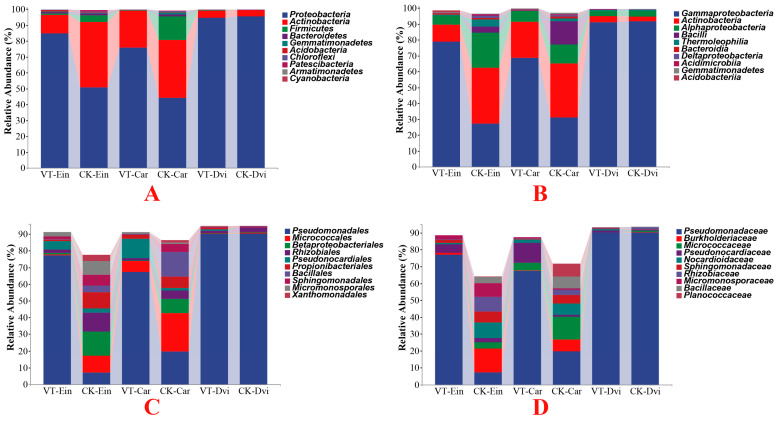
Cumulative bar charts of the relative abundance of taxa (top 10) at the phylum (**A**), class (**B**), order (**C**), and family (**D**) levels. VT-Dvi, *D. viscosa* in the VTM mining area; VT-Car, *Carex* in the VTM mining area; VT-Ein, *E. indica* in the VTM mining area; CK-Dvi, *D. viscosa* in the non-VTM mining area; CK-Car, *Carex* in the non-VTM mining area; CK-Ein, *E. indica* in the non-VTM mining area.

By examining the abundance of the top 10 phyla, differences among the samples were assessed (Figure 2A). *Proteobacteria*, the dominant phylum in the samples, was found to make up an average of 74.36% of the total bacteria, followed by *Actinobacteria* (20.19%), *Firmicutes* (3.44%), and *Bacteroidetes* (0.63%). Among all the samples, *Proteobacteria* (95.61% on average) was the predominant phylum in the CK-Dvi sample, *Actinobacteria* (41.23% on average) dominated the CK-Ein sample, while *Firmicutes* (14.83% on average) and *Bacteroidetes* (1.35% on average) were the most abundant in the CK-Car sample. Compared to the CK-Ein sample, there was a notable boost in the abundance of *Proteobacteria* within the VT-Ein samples (*p* < 0.05), whilst a considerable decrease in the quantity of *Actinobacteria* was observed (*p* < 0.05). Besides, the abundances of *Firmicutes* and *Bacteroidetes* decreased, but not significantly. A notable change was observed in the abundance of *Proteobacteria* in the VT-Car sample, which demonstrated a significant rise compared to the CK-Car sample (*p* < 0.05). Furthermore, the abundance of *Bacteroidetes* in the VT-Car sample was significantly reduced compared to the CK-Car sample (*p* < 0.05). Besides, the abundances of *Actinobacteria* and *Firmicutes* were decreased in the VT-Car sample, but not significantly. While a drop in the abundance of *Proteobacteria* was observed in the VT-Dvi sample compared to the CK-Dvi sample, this difference was not found to be statistically significant. Additionally, the abundances of *Actinobacteria*, *Firmicutes*, and *Bacteroidetes* were increased, but not significantly.

At the class level, *Gammaproteobacteria* predominated in all samples, comprising an average of 64.82%, with *Actinobacteria* following at 18.23%, *Alphaproteobacteria* at 8.87%, and *Bacilli* at 3.24% (Figure 2B). Compared to the CK-Ein sample, the VT-Ein samples exhibited a significantly increased abundance of *Gammaproteobacteria* (*p* < 0.05), a notably decreased abundance of *Actinobacteria* and *Alphaproteobacteria* (*p* < 0.05), and a reduced abundance of *Bacilli*, but not significantly. In the VT-Car sample, it was noted that the relative abundance of *Gammaproteobacteria* significantly increased compared to the CK-Car sample (*p* < 0.05), while the abundances of *Actinobacteria*, *Alphaproteobacteria*, and *Bacilli* all showed decreases, although not statistically significant. In the VT-Dvi sample, the abundance of *Actinobacteria* was increased compared to the CK-Dvi sample, but not significantly. Additionally, the abundances of *Gammaproteobacteria*, *Alphaproteobacteria*, and *Bacilli* were decreased, but not significantly.

At the order level, members of the *Pseudomonadales* were found to be the most prevalent with an average abundance of 58.62%, followed by *Micrococcales* (6.77%), *Betaproteobacteriales* (4.17%), and *Rhizobiales* (3.98%) (Figure 2C). In comparison to the CK-Ein sample, the VT-Ein sample showed a notably higher abundance of *Pseudomonadales* (*p* < 0.05). Additionally, the abundances of *Betaproteobacteriales* and *Rhizobiales* exhibited a significant decrease (*p* < 0.05), while the abundance of *Micrococcales* also decreased, but not significantly. In the VT-Car sample, the abundance of *Pseudomonadales* showed a significant increase compared to the CK-Car sample (*p* < 0.05). A significant decrease in the abundance of *Betaproteobacteriales* was observed (*p* < 0.05), with the abundances of *Micrococcales* and *Rhizobiales* also showing decreases, although not statistically significant. Compared to the CK-Dvi sample, the abundances of *Pseudomonadales*, *Betaproteobacteriales*, *Micrococcales*, and *Rhizobiales* in the VT-Dvi sample were all reduced, but not significantly.

At the family level, *Pseudomonadaceae* had the highest proportion, representing an average of 58.62% of the total population in the samples, with *Burkholderiaceae* (3.81%), *Micrococcaceae* (3.74%), and *Pseudomonocardiaceae* (3.63%) following in abundance (Figure 2D). In comparison to the CK-Ein sample, the VT-Ein sample exhibited a significantly higher abundance of *Pseudomonadaceae* (*p* < 0.05). Furthermore, the abundance of *Burkholderiaceae* was notably lower (*p* < 0.05), while the abundance of *Micrococcaceae* was also lower, but not significantly. Additionally, the abundance of *Pseudomonocardiaceae* was greater, although not statistically significant. In comparison to the CK-Car sample, the VT-Car sample exhibited a significantly greater abundance of *Pseudomonadales* (*p* < 0.05). The overall abundance of *Burkholderiaceae* decreased notably (*p* < 0.05), whereas the decrease in *Micrococcaceae* was not statistically significant. Moreover, the prevalence of *Pseudomonocardiaceae* was higher, although not statistically significant. Compared to the CK-Dvi sample, the abundances of *Pseudomonadaceae*, *Burkholderiaceae*, and *Micrococcaceae* in the VT-Dvi sample were all decreased, but not significantly, while the abundance of *Pseudonocardiaceae* was increased, but not significantly.

**Figure 3:**
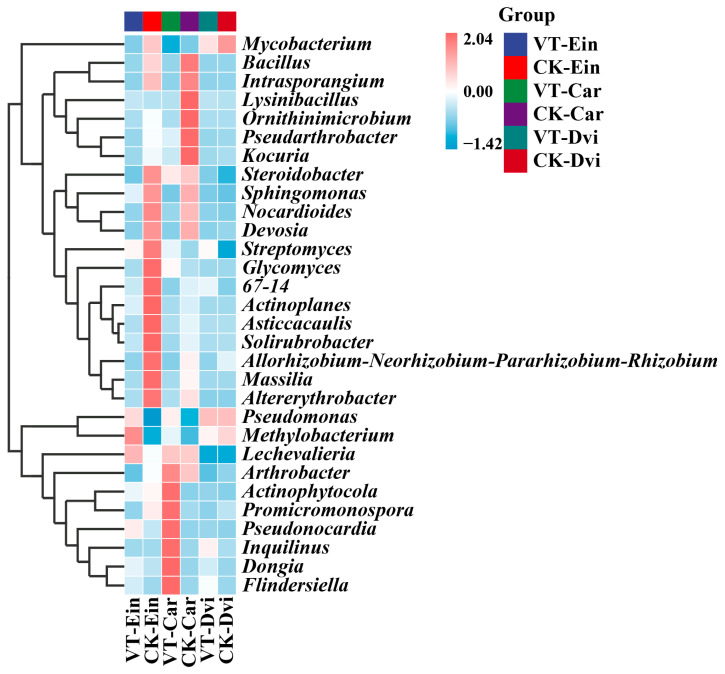
Heatmap analysis of the 30 genes with the highest abundance of different taxa samples. The relative abundances of different genera are represented by different colors. Blocks, with colors ranging from blue to red indicate an increase in relative abundance. VT-Dvi, *D. viscosa* in the VTM mining area; VT-Car, *Carex* in the VTM mining area; VT-Ein, *E. indica* in the VTM mining area; CK-Dvi, *D. viscosa* in the non-VTM mining area; CK-Car, *Carex* in the non-VTM mining area; CK-Ein, *E. indica* in the non-VTM mining area.

At the genus level, *Pseudomonas* emerged as the most prevalent, representing an average of 58.60% of the total bacteria in the samples, followed by *Nocardioides* (2.90%), *Pseudonocardia* (2.44%), and *Pseudarthrobacter* (1.94%) (Figure 3). Compared to the CK-Ein sample, the VT-Ein sample showed a significantly increased abundance of *Pseudomonas* (*p* < 0.05) and a significantly reduced abundance of *Nocardioides* (*p* < 0.05). The abundance of *Pseudonocardia* was also increased, but not significantly. Additionally, the abundance of *Pseudarthrobacter* was decreased, but not significantly. In comparison to the CK-Car samples, there was a significant increase in the abundance of *Pseudomonas* in the VT-Car sample (*p* < 0.05). Moreover, there was a notable decrease in the amount of *Nocardioides* (*p* < 0.05), while the abundance of *Pseudonocardia* increased, but not significantly. The abundance of *Pseudarthrobacter* exhibited a decrease, although it was not statistically significant. Compared to the CK-Dvi sample, the abundances of *Pseudomonas* and *Pseudarthrobacter* were both reduced, but not significantly, while the abundances of *Nocardioides* and *Pseudonocardia* were increased, but not significantly.

### 3.4. Structural Differentiation of Endophytic Bacterial Communities

**Figure 4:**
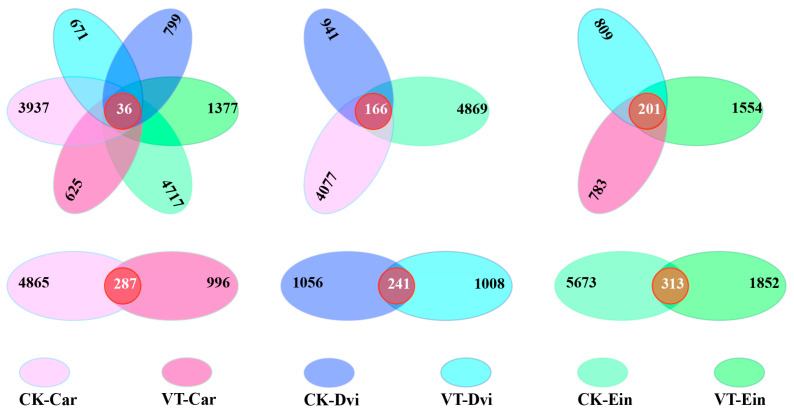
Differential microbial comparison between different samples. VT-Dvi, *D. viscosa* in the VTM mining area; VT-Car, *Carex* in the VTM mining area; VT-Ein, *E. indica* in the VTM mining area; CK-Dvi, *D. viscosa* in the non-VTM mining area; CK-Car, *Carex* in the non-VTM mining area; CK-Ein, *E. indica* in the non-VTM mining area.

An examination of the distinct OTUs and the OTUs shared among the samples indicated the presence of 36 core OTUs in all samples. Additionally, there were 4717, 1377, 3937, 625, 799, and 671 OTUs specific to CK-Ein, VT-Ein, CK-Car, VT-Car, CK-Dvi, and VT-Dvi samples, respectively (Figure 4). Among the three types of plant root systems in the VTM mining area, the VT-Ein, VT-Car, and VT-Dvi samples had 1554, 783, and 809 specific OTUs and 201 shared OTUs, respectively. Among the three plant root systems in the non-VTM mining area, the CK-Ein, CK-Car, and CK-Dvi samples had 4869, 4077, and 941 specific OTUs and 166 shared OTUs, respectively. Between the CK-Ein and VT-Ein samples, the CK-Ein sample contained 5673 specific OTUs, while the VT-Ein sample contained 1852 specific OTUs. There were 313 common OTUs between the two samples. Among the CK-Car and VT-Car samples, the CK-Car sample contained 4865 specific OTUs, while the VT-Car sample contained 996 specific OTUs. There were 287 common OTUs between the two samples. Between the CK-Dvi and VT-Dvi samples, the CK-Dvi sample contained 1056 specific OTUs, while the VT-Dvi sample contained 1008 specific OTUs. There were 241 common OTUs between the two samples.

**Figure 5:**
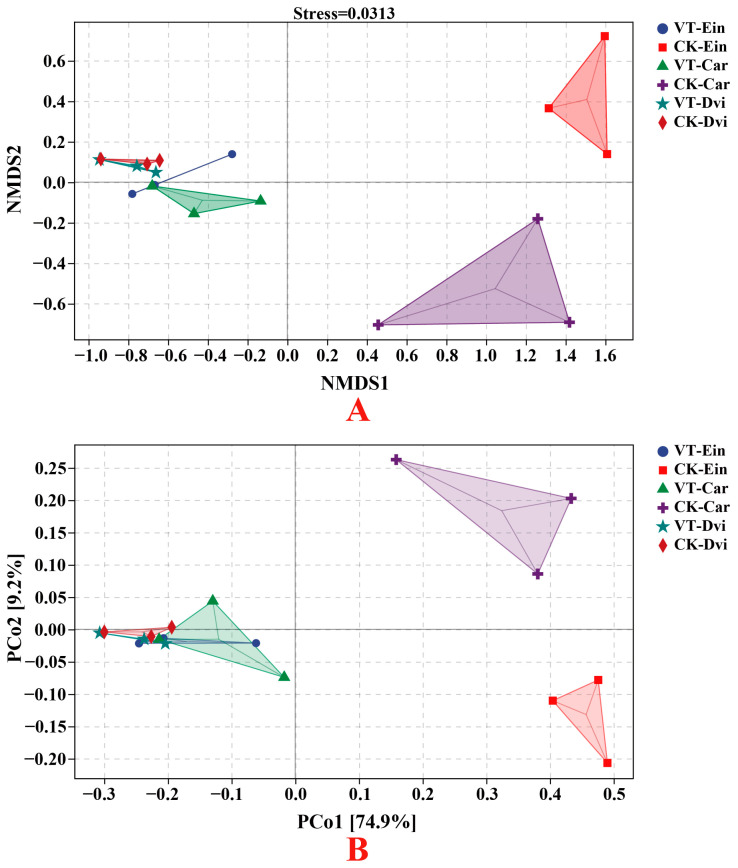
β diversity analysis of the root endophytic bacterial community in different samples based on NMDS (**A**) and PCoA (**B**). VT-Dvi, *D. viscosa* in the VTM mining area; VT-Car, *Carex* in the VTM mining area; VT-Ein, *E. indica* in the VTM mining area; CK-Dvi, *D. viscosa* in the non-VTM mining area; CK-Car, *Carex* in the non-VTM mining area; CK-Ein, *E. indica* in the non-VTM mining area.

We employed NMDS and PCoA to assess variations in endophytic bacterial communities within the root systems across different samples (Figure 5). The results indicated that mining VTM resulted in variations in the composition of the endophytic bacterial community within the root systems of *Carex* and *E. indica* when compared to areas without VTM mining. In summary, the NMDS and PCoA analyses conducted on the bacterial communities present in the roots of *Carex* and *E. indica* demonstrated that the VTM mining activities significantly altered the overall structure and composition of the endophytic bacterial community. However, there was no significant difference in the community composition of endophytic bacteria in the root systems of *D. viscosa* between VTM mining and non-VTM mining areas. This suggests that, for endophytic bacteria in the root system of *D. viscosa*, internal environmental factors were more influential than external environmental factors.

### 3.5. Function Prediction of the Endophytic Bacterial Communities

**Figure A2:**
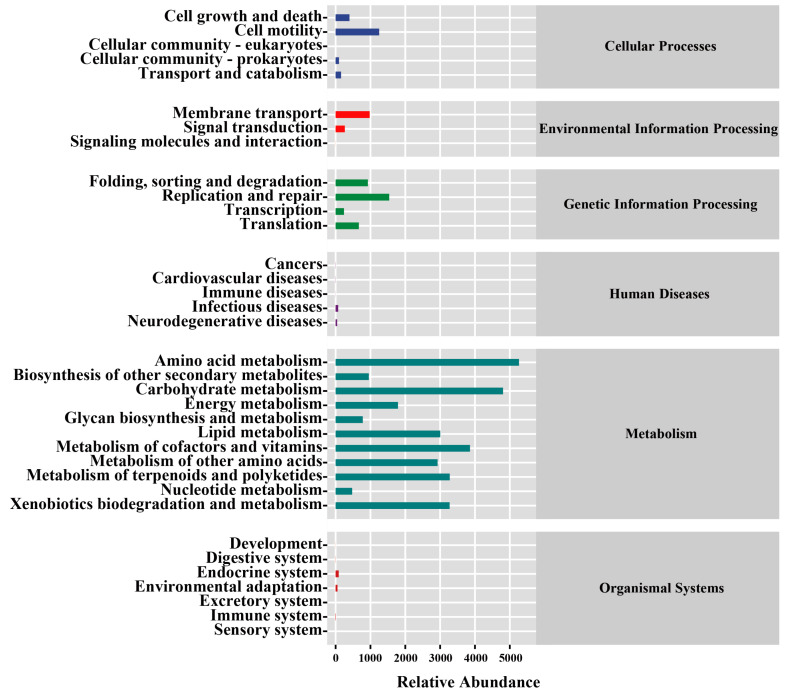
Functional enrichment analysis based on the KEGG predicted by PICRUSt2.

**Figure A3:**
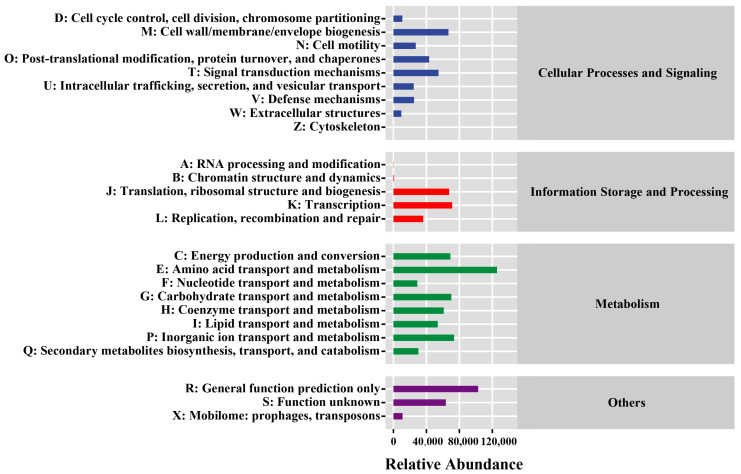
Functional enrichment analysis based on the COG predicted by PICRUSt2.

**Figure A4:**
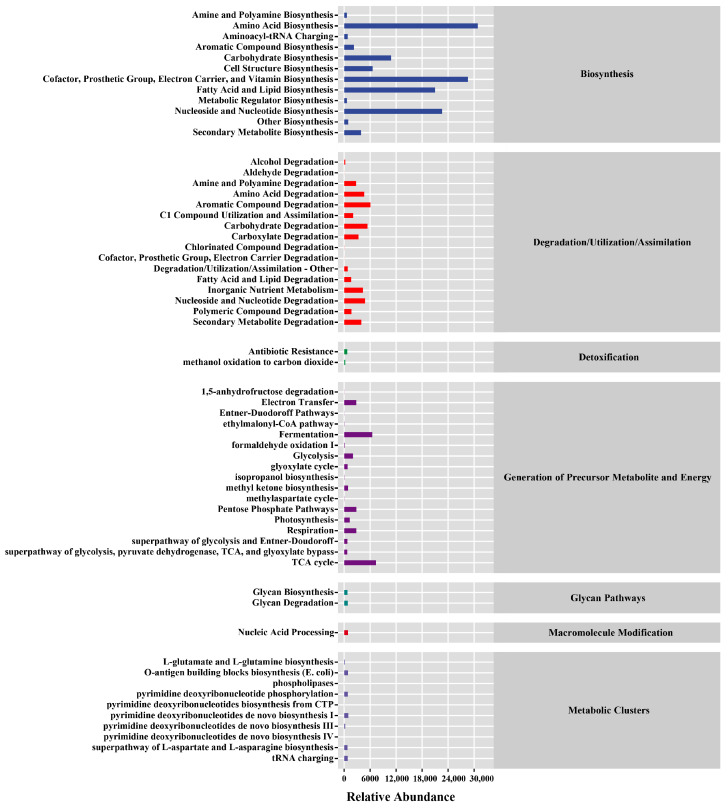
Functional enrichment analysis based on the MetaCyc database predicted by PICRUSt2.

PICRUSt2 was employed to gain a thorough understanding of the specific functions of the root endophytic bacteria present in the samples. According to the data in the KEGG database, the various genes found in bacterial cells have been organized into six broad categories (Figure A2). The largest group of these genes can be categorized as metabolism genes, which were found to represent 81.78% of all the genes in the database. The second largest group, representing 9.06% of the total genes, can be categorized as genetic information processing genes, followed by cellular processes genes, which represent 5.10% of the total. At the second level, amino acid metabolism, carbohydrate metabolism, metabolism of cofactor and vitamins, xenobiotics biodegradation, and metabolism of terpenoids and polyketides were the most abundant and accounted for 14.16%, 12.93%, 10.37%, 8.82%, and 8.80%, respectively. Based on the COG database, approximately 84.28% of bacterial genes were categorized into three groups (Figure A3). Metabolism stood out as the most enriched function, encompassing 45.43% of the genes, while cellular process and signaling accounted for 23.23%, and information storage and processing for 18.52%. Second-level COG metabolism showed that genes involved in amino acid transport and metabolism, transcription, inorganic ion transport and metabolism, and carbohydrate transport and metabolism were the most abundant, accounting for 11.14%, 6.53%, 6.32%, and 6.23%, respectively. According to the MetaCyc database, bacterial genes can be divided into seven categories (Figure A4). In the observed data, it can be seen that biosynthesis is the most prevalent category among the samples, representing a staggering 62.15% of the total. The second most common category is degradation/utilization/assimilation, accounting for 20.04%, while the third is the generation of precursor metabolites and energy, making up 13.91% of all samples. In the second level, the largest proportion of genes was found in amino acid biosynthesis (14.79%), followed by cofactors, prosthetic groups, electron carrier, and vitamin biosynthesis (13.71%), nucleoside and nucleotide biosynthesis (10.85%), fatty acid and lipid biosynthesis (10.07%), and carbohydrate biosynthesis (5.19%).

**Figure 6:**
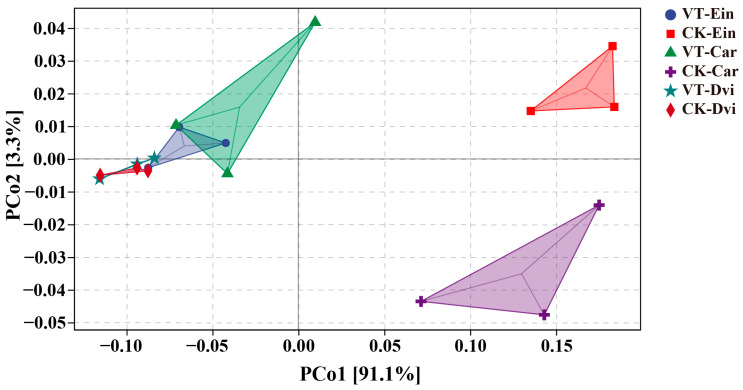
PCoA of the functions of the root endophytic bacteria predicted by PICRUSt2. VT-Dvi, *D. viscosa* in the VTM mining area; VT-Car, *Carex* in the VTM mining area; VT-Ein, *E. indica* in the VTM mining area; CK-Dvi, *D. viscosa* in the non-VTM mining area; CK-Car, *Carex* in the non-VTM mining area; CK-Ein, *E. indica* in the non-VTM mining area.

We employed PCoA to evaluate variations in endophytic bacterial function across different samples, as illustrated in Figure 6. The findings indicated that endophytic bacteria in the root systems of *Carex* and *E. indica* exhibited variations when subjected to VTM mining activities, as opposed to those from non-VTM mining areas. Moreover, the mineral mining operations conducted by VTM resulted in a convergence of the functionalities of endophytic bacteria within the root structures of these three plant species.

### 3.6. Functional Enrichment of Endophytic Bacteria

**Figure 7:**
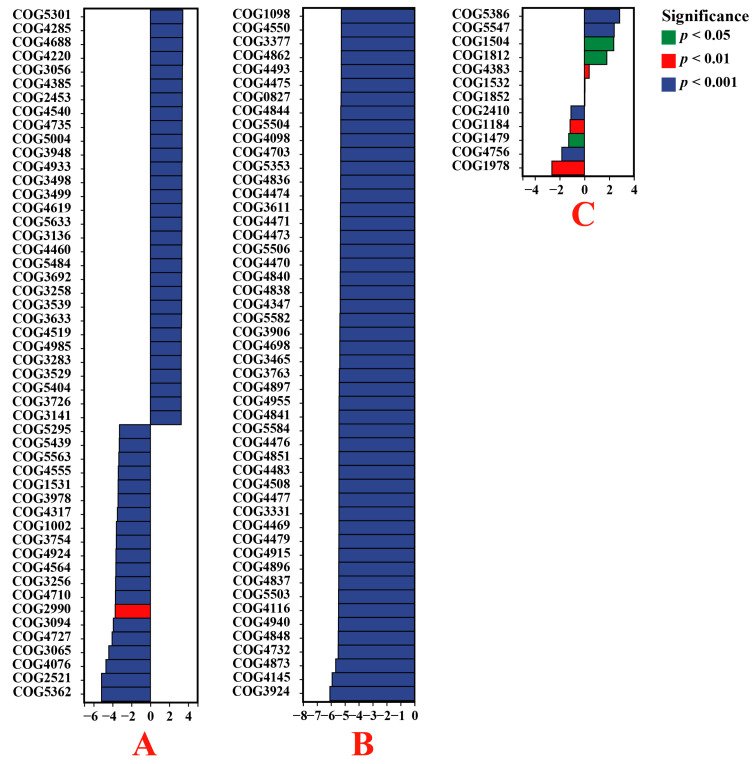
Based on the COG database, the functions of endophytic bacteria in plant roots were significantly different between VTM mining areas and non-VTM mining areas. The vertical axis represents the ID of the COG, the horizontal axis represents the log2-fold change, and different colors indicate significant differences between samples at different levels. (**A**) VT-Ein vs. CK-Ein; (**B**) VT-Car vs. CK-Car; (**C**) VT-Dvi vs. CK-Dvi.

The analysis of the COG database revealed that the endophytic bacteria present in the roots of *E. indica* from the VTM mining area contained significantly increased levels of phage-related tail fiber protein (COG5301), uncharacterized protein (COG4285), uncharacterized protein (COG4688), ohage DNA packaging protein (COG4220), and uncharacterized protein (COG3056) compared to those from the non-VTM mining area (*p* < 0.05). In contrast, the levels of the phage terminase large subunit (COG5362), predicted archaeal methyltransferase (COG2521), predicted RNA methylase (COG4076), starvation-inducible outer membrane lipoprotein (COG3065), and uncharacterized protein (COG4727) were notably reduced (*p* < 0.05) (Figure 7). The levels of uncharacterized protein (COG3924), Na+/panthothenate symporter (COG4145), uncharacterized protein (COG4873), predicted membrane protein (COG4732), and uncharacterized protein (COG4848) in the endophytic bacteria of the *Carex* root system from the VTM mining area were significantly lower than those in the non-VTM mining area (*p* < 0.05). Compared to *D. viscosa* from the non-VTM area, the endophytic bacteria in the root systems of *D. viscosa* from the VTM mining area had higher levels of the heme-binding NEAT domain (COG5386), uncharacterized protein (COG5547), uncharacterized protein (COG1504), archaeal S-adenosylmethionine synthetase (COG1812), and Mu-like prophage protein gp29 (COG4383), which were significantly reduced (*p* < 0.05). In contrast, the contents of predicted RNase H-related nuclease YkuK, DUF458 family (COG1978), predicted cation transporter (COG4756), uncharacterized protein (COG1479), translation initiation factor 2B subunit, eIF-2B α/β/delta family (COG1184), and predicted nuclease (RNAse H fold) (COG2410) were significantly reduced (*p* < 0.05).

**Figure 8:**
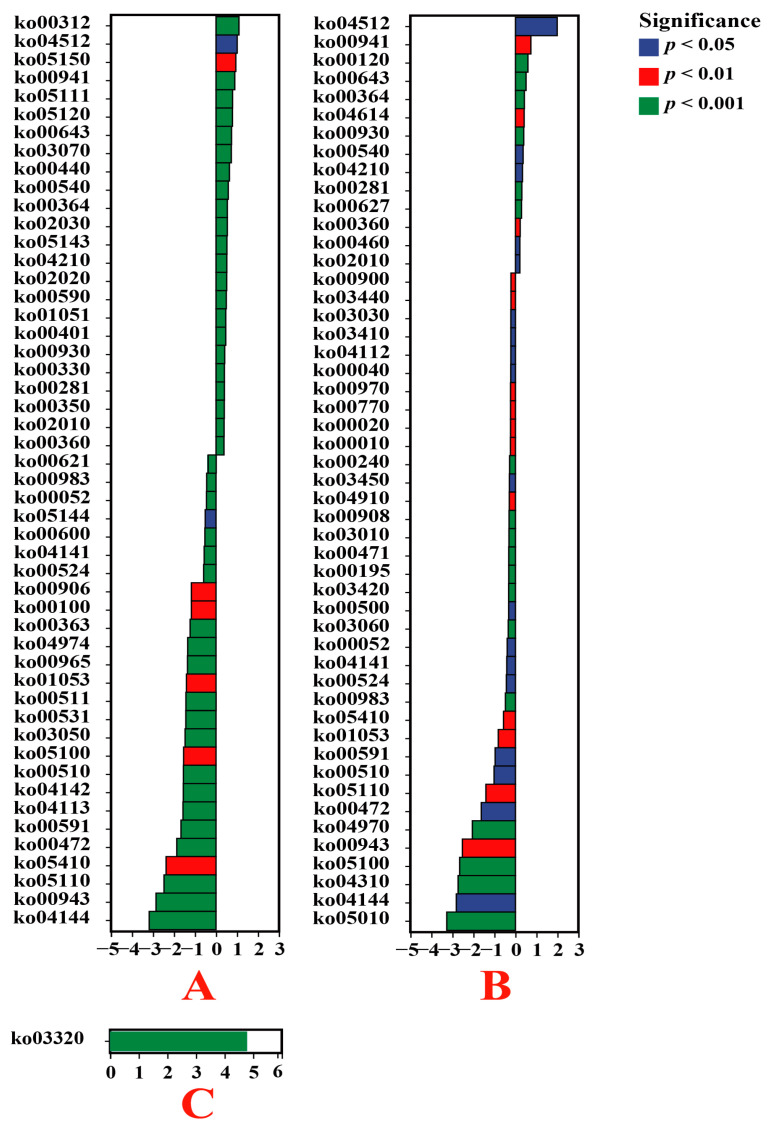
Based on the KO database, the functions of endophytic bacteria in plant roots were significantly different between VTM mining areas and non-VTM mining areas. The vertical axis represents the ID of the KO, the horizontal value is prelog2 (fold change), and different colors indicate significant differences between samples at different levels. (**A**) VT-Ein vs. CK-Ein; (**B**) VT-Car vs. CK-Car; (**C**) VT-Dvi vs. CK-Dvi.

Owing to the impact of VTM extraction operations, the endophytic bacteria in the root systems of *E. indica* from the mining area included β-Lactam resistance (ko00312), ECM-receptor interaction (ko04512), *Staphylococcus aureus* infection (ko05150), flavonoid biosynthesis (ko00941), and Vibrio cholerae pathogenic cycle (ko05111), which significantly increased (*p* < 0.05). Meanwhile the content of endocytosis (ko04144), isoflavonoid biosynthesis (ko00943), Vibrio cholerae infection (ko05110), hypertrophic cardiomyopathy (ko05410), and D-arginine and D-ornithine metabolism (ko00472) significantly decreased (*p* < 0.05) (Figure 8). The endophytic bacteria of the *Carex* root system from the VTM mining area had significantly higher amounts of ECM-receptor interaction (ko04512), flavonoid biosynthesis (ko00941), primary bile acid biosynthesis (ko00120), styrene degradation (ko00643), and fluorobenzoate degradation (ko00364) than those from the non-VTM mining region (*p* < 0.05). Meanwhile, the abundances of genes involved in Alzheimer’s disease (ko05010), endocytosis (ko04144), Wnt signaling pathway (ko04310), bacterial invasion of epithelial cells (ko05100), and isoflavonoid biosynthesis (ko00943) were notably lower (*p* < 0.05). Compared to *D. viscosa* from the non-VTM mining area, only the content of the PPAR signaling pathway (ko03320) was significantly increased among endophytic bacteria in the root system of *D. viscosa* from the VTM mining area (*p* < 0.05). Conversely, the degree of ECM-receptor interaction (ko04512) and flavonoid biosynthesis (ko00941) was visibly augmented in root-dwelling endophytic bacteria of *E. indica* and *Carex* from the vicinity of VTM mining operations when contrasted with their counterparts unassociated with mining activities (*p* < 0.05). However, the levels of endocytosis (ko04144), isoflavonoid biosynthesis (ko00943), and Alzheimer’s disease (ko05010) in root endophytic bacteria of *E. indica* and *Carex* from the VTM mining compared to non-VTM mines were significantly reduced (*p* < 0.05).

**Figure 9:**
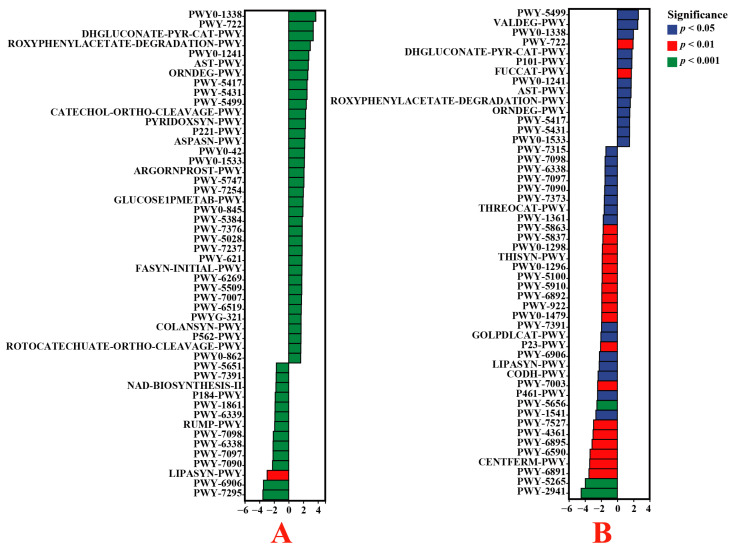
Based on the MetaCyc database, the functions of endophytic bacteria in plant roots were significantly different between VTM mining areas and non-VTM mining areas. The vertical axis represents the ID of the pathway, the value of pre-axis log2 (fold change), and different colors indicate significant differences between samples at different levels. (**A**) VT-Ein vs. CK-Ein; (**B**) VT-Car vs. CK-Car.

According to MetaCyc analysis, it was indicated that the functions of polymyxin resistance (PWY0-1338), nicotinate degradation I (PWY-722), glucose degradation (oxidative) (DHGLUCONATE-PYR-CAT-PWY), ADP-L-glycero-β-D-manno-heptose biosynthesis (PWY0-1241), and 4-hydroxyphenylacetate degradation (3-HYDROXYPHENYLACETATE-DEGRADATION-PWY) were significantly enhanced in the root bacteria of endophytic *E. indica* from the VTM mining area compared to those from the non-VTM mining region (*p* < 0.05). Additionally, the levels of L-arabinose degradation IV (PWY-7295), chitin-derivative degradation (PWY-6906), phospholipases (LIPASYN-PWY), UDP-2,3-diacetamido-2,3-dideoxy-α-D-mannuronate biosynthesis (PWY-7090), and vanillin and vanillate degradation I (PWY-7097) were significantly lower (*p* < 0.05) (Figure 9). In comparison with the non-VTM mining area, the endophytic bacterial population within *Carex* roots within the confines of the VTM mining area contained significantly increased levels of vitamin B6 degradation (PWY-5499), L-valine degradation I (VALDEG-PWY), polymyxin resistance (PWY0-1338), nicotinate degradation I (PWY-722), and glucose degradation (oxidative) (DHGLUCONATE-PYR-CAT-PWY) (*p* < 0.05). On the other hand, L-lysine biosynthesis II (PWY-2941), peptidoglycan biosynthesis II (staphylococci) (PWY-5265), thiazole biosynthesis II (Bacillus) (PWY-6891), the superpathway of Clostridium acetobutylicum acidogenic fermentation (PWY-6590), and pyruvate fermentation to butanoate (CENTFERM-PWY) were significantly reduced (*p* < 0.05). Nevertheless, the metabolic pathways associated with endophytic bacteria in the roots of *D. viscosa* exhibited no discernible disparity between the VTM mining area and the non-VTM mining area. In addition, compared to the non-VTM mining area, polymyxin resistance (PWY0-1338), nicotinate degradation I (PWY-722), and glucose degradation (oxidative) (DHGLUCONATE-PYR-CAT-PWY) were significantly higher in the endophytic bacteria in the roots of *E. indica* and *Carex* from the VTM mining area.

### 3.7. Correlation Analysis of the Endophytic Bacteria Communities

**Figure 10:**
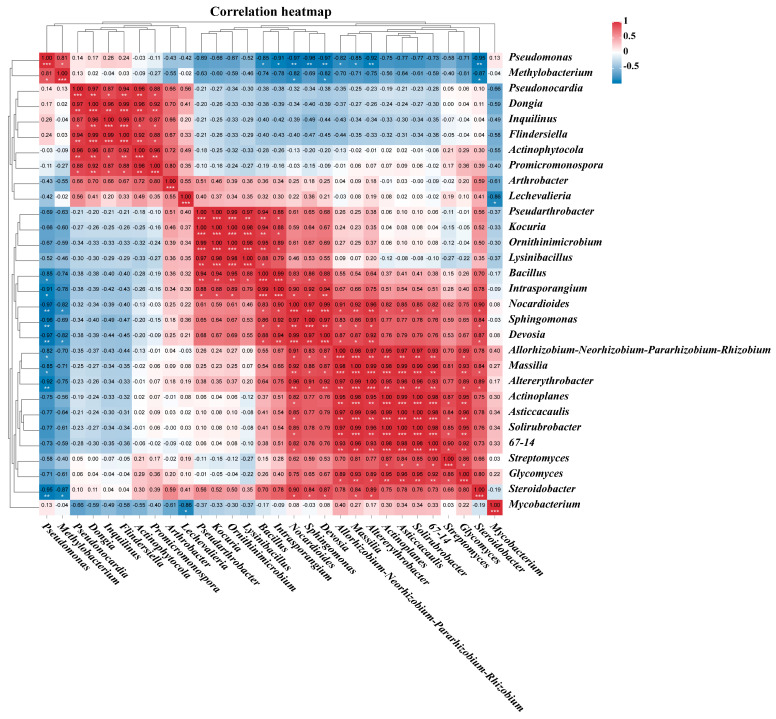
Pearson correlation analysis between the 30 genes with the highest abundance in different samples. The Pearson correlation index is marked in a square color block, with a blue color block indicating a negative correlation between two bacterial genera and a red color block indicating correlation. One asterisk indicates a significant correlation between two bacterial genera at the *p* < 0.05 level, two asterisks indicate a significant correlation between two bacterial genera at the *p* < 0.01 level, and three asterisks indicate a significantly greater correlation at the *p* < 0.001 level.

Pearson correlation analysis revealed significant interactions among the 30 most abundant genera of plant root endophytic bacteria from the VTM mining area (Figure 10). Positive correlations were observed between *Altererythrobacter*, *Actinoplanes*, *Asticcacaulis*, *Solirubrobacter*, *67–14*, and *Nocardioides* (*p* < 0.05). Moreover, *Dongia*, *Inquilinus*, *Flindersiella*, *Actinophytocola*, *Promicromonospora*, and *Pseudonocardia* were also found to be positively correlated (*p* < 0.05). On the other hand, negative correlations were observed between *Bacillus*, *Intrasporangium*, *Nocardioides*, *Sphingomonas*, *Devosia*, *Allorhizobium-Neorhizobium-Pararhizobium-Rhizobium*, *Massilia*, *Altererythrobacter*, and *Pseudomonas*. Additionally, *Nocardioides*, *Devosia*, *Steroidobacter*, and *Methylobacterium* were also negatively correlated (*p* < 0.05). The study illustrated a significant interplay evident among the primary cohorts of endophytic bacterial communities active within plant root systems.

## 4. Discussion

VTM is an important mineral resource, and with increasing market demand, the mining scale of VTM is also constantly expanding. Nevertheless, significant VTM tailings are created when mining VTM. Tailings are frequently disposed of in dams, leading to the potential release of toxic pollutants into the surrounding environment, thereby endangering human health [17,18,19]. In China, Panzhihua City is a major mining area for VTM [52]. Therefore, we collected three types of plants near the mining area to study the ecological effects of mining activities. Although existing studies have analyzed the characteristics of microbial communities in the soil of the VTM mining area [53], no study has demonstrated a relationship between VTM mining operations and the diversification or population dynamics of endophytic bacterial communities within the plant roots adjacent to such operations. Endophytes are crucial for enhancing plant growth, development, stress resilience, and defense against pests and diseases, and some endophytes can also protect plants from heavy metals [54,55,56]. However, reductions in the richness of endophytes can elicit diminished plant attributes such as quality and resilience under stress [57]. Consequently, the potential impact of VTM mining on the biodiversity of endophytic bacteria within nearby plants necessitates careful evaluation. In this investigation, specific focus has been placed on three indigenous plants in close proximity to the VTM mining area, revealing the ramifications of VTM mining on the endophytic bacterial populations and functional activities within the root systems of these three plant species. These findings significantly augment our comprehension of the ecological implications of VTM mining activities.

### 4.1. Microbial Community Responses

Jose et al. demonstrated that increased levels of heavy metals result in alterations to the diversity of soil bacterial communities, which is consistent with our findings [58]. Our study showed that VTM mining activities directly led to a significant decrease in endophytic bacterial diversity and community richness indicators in the root systems of *E. indica* and *Carex* in the mining area. This could be attributed to the loss of soil nutrients caused by mining activities, resulting in a decline in the quality of living conditions for microbes and ultimately leading to a decrease in microbial diversity [53]. This result indicates that the negative impact of VTM mining on the endophytic bacteria in the roots of nearby plants may be due to the diffusion of harmful components, such as heavy metals, and alterations in soil physicochemical characteristics induced by VTM mining activities. Further study revealed that VTM mining activities resulted in the differentiation of endophytic bacterial communities within the roots of *E. indica* and *Carex* in the mining area. Therefore, the ecological impact of VTM mining deserves widespread attention. Furthermore, we observed lower abundances of *Nocardioides* and *Pseudarthrobacter* in the roots of *E. indica* and *Carex* from the vicinity of the VTM mining site compared to those present in the non-VTM mining region. The findings uncovered the susceptibility of these two strains to environmental alterations instigated by VTM mining. We detected *Proteobacteria*, *Actinobacteria*, *Firmicutes*, and *Bacteroidetes* in our samples, which is similar to the findings of Zhang et al. and Tang et al. that these bacteria are widespread in contaminated environments [59,60]. Although environmental contamination from mining activities can severely damage the microecology of native plants, there are microorganisms that have been able to adapt to these adverse conditions under prolonged heavy metal stress [61]. These microorganisms have developed a variety of anti-stress strategies to increase their tolerance and ultimately mitigate the effects of toxicity [62,63]. Remarkably, we observed an enrichment of *Pseudomonas* within the root structures of *E. indica* and *Carex* growing in the vicinity of the VTM mining area. This is similar to the findings of Tang et al., Xiao et al., and Zhang et al., where the phylum *Pseudomonas* consistently constitutes the most dominant phylum in environments contaminated with heavy metals [60,64,65]. In addition, the impact of mining activities led to a significant increase in the relative abundance of *Pseudomonas* in the root systems of *E. indica* and *Carex*. This suggests that *Pseudomonas* is well adapted to environments contaminated with heavy metals, and its extreme adaptability to heavy metals can be explained by its efficient utilization of resources and metabolic activity [66,67]. It has been shown that *Pseudomonas* can reduce highly toxic pentavalent V to less toxic tetravalent V under certain conditions, thereby reducing its toxicity [67]. At the same time, some studies have shown that *Pseudomonas* has a certain resistance to heavy metals and is widely used in heavy metal remediation [68,69,70,71]. Additionally, *Actinobacteria* and *Firmicutes* were dominant among the root endophytes in these samples, which could be attributed to their high tolerance to heavy metals. According to Pan et al., *Actinobacteria* were found to be effectively enriched in heavy metal-contaminated soils, dominating all of the microbial communities [72]. Meanwhile, the study by Wang et al. found that *Firmicutes* could also play a role in reducing V to decrease exposure to toxicity. In addition, other dominant microbiota may also be involved in the reduction of V, since V is also a metal that is easily reduced by oxidation [73]. Therefore, these bacteria can be further developed and utilized for bioremediation in VTM mining areas. The abundance of two bacteria, *Proteobacteria* and *Actinobacteria*, accounted for a significant portion of the sum of all microbial communities in the samples, which may be due to their unique ability to bind heavy metals, allowing them to adapt to harsh heavy metal-contaminated environments [74,75,76].

Hierarchical taxonomic descriptions at the genus level allow for further analysis of the more complex details of bacterial community structure. At this level of genus, *Pseudomonas*, *Nocardioides*, *Pseudonocardia*, and *Pseudarthrobacter* are the dominant microbial communities. It has been shown that these bacteria have good resistance to heavy metals, that they coexist well with heavy metals, and that these bacteria are often isolated in a variety of environments heavily contaminated with heavy metals [72,77,78]. In addition, other studies have shown that *Pseudarthrobacter* is not only enriched in heavy metal-contaminated environments, but also plays a key role in promoting plant growth, and therefore, this bacterium also has great potential for application in bioremediation at VTM mining sites [79,80]. The strong resistance of these bacteria and their high tolerance to heavy metals have led to their complete dominance among the endophytic bacteria in the root systems of plants in VTM mining areas.

Furthermore, our study also revealed that the levels of enrichment of endophytic bacteria within plant roots in the VTM mining area were different, indicating that plants have certain screening and shaping abilities for endophytic bacterial communities. Throughout the extended coexistence and integration of endophytic bacteria within the plant root system, only competitive and adaptive bacteria are able to successfully colonize and form an advantageous mutualistic association with the host organism [81].

Overall, our study revealed that VTM mining significantly influenced the distribution of endophytic bacterial communities in the root systems of *E. indica* and *Carex* within the mining region. Under contaminated environmental conditions, the microbial communities in *E. indica* and *Carex* roots in VTM mining areas consist of heavy metal-resistant bacteria, which is the result of long-term selection by plants. Studying changes in microbial communities due to mining activities can help us identify bacteria with good environmental adaptations to aid in the bioremediation of mining sites.

### 4.2. Functional Enrichment Analysis

We also used PICRUSt2 to infer the function of the endophytic bacterial community in plant roots through three databases. We found that VTM mining also caused the development of distinct functions in the endophytic bacterial community in plant roots. Plants in VTM mining areas have different enriched microbial community functions compared to plants in non-VTM mining areas. The VTM mining has a profound impact on the functionality of endophytic bacteria within plant root systems prevalent in VTM mining regions. For example, the abundance of the phage-related tail fiber protein (COG5301), β-Lactam resistance (ko00312), and ECM-receptor interaction (ko04512) functions significantly augmented (*p* < 0.05). These functional changes revealed the local response mechanisms of endophytic bacteria in plant roots to VTM mining and environmental stress.

Jacquiod et al., found a high percentage of phage-related mRNA sequences in mRNAs in soils chronically contaminated with Cu, which is similar to the results we found [82]. We suggest that in the root systems of plants in the VTM mining area, their endophytic communities may maintain the stability of each function of the community by increasing the expression of the phage-related tail fiber protein (COG5301) function. The up-regulation of this function may potentially play a role in the survival of these microbial communities. Some studies have shown that β-Lactam is a widely used antibiotic, which is commonly used in the treatment of bacterial infections [83]. The functions of heavy metals and β-Lactam have some similarities, and they are both effective in inhibiting bacterial growth [84]. In VTM mining areas, mining activities can lead to elevated levels of heavy metals in nearby soil, posing a great challenge for the survival of local plants and microorganisms. Therefore, in the function of plant rhizosphere endophytes in the VTM mining area, microbial communities may reduce the repressive function caused by heavy metals by increasing the expression of β-Lactam resistance (ko00312) function, which enables them to survive in the heavy metal-exposed environment. Meng et al. found a significant enrichment of the ECM-receptor interaction (ko04512) function in the digestive glands of Cu-exposed Japanese scallops [85]. Similarly, Yao et al., also found a significant enrichment of this function in the livers of antimony-exposed zebrafish [86]. Our results were similar to theirs; the expression of the ECM-receptor interaction (ko04512) function was also significantly elevated as a function of the endophytic bacterial community in plant roots in the VTM mining area. ECM plays an important role in maintaining the structure and function of the cell [87]. Therefore, endophytic bacteria in the root systems of plants in the VTM mining area may ensure the function of the microbial community by enhancing the expression of the ECM-receptor interaction (ko04512) function. In conclusion, our study found that endophytic bacteria in the root systems of plants in the VTM mining area adapt to environmental changes by modulating various functions.

Furthermore, it was determined for the first time that the community structure and functional role of the endophytic bacteria within the roots of *D. viscosa* remained relatively unaffected between the VTM mining area and the non-VTM mining area. This indicates that *D. viscosa* itself has a strong adaptability to environmental changes. Therefore, it is also possible to consider using *D. viscosa* for the ecological restoration of VTM mining regions.

## 5. Conclusions

This study examined the impact of VTM mining on the diversity, community composition, and metabolic function of endophytic bacteria in the roots of *E. indica*, *Carex*, and *D. viscosa*. The study findings indicate that VTM mining negatively impacted the biodiversity of endophytic bacteria in the root systems of *E. indica* and *Carex* in the mining area. The Chao1, observed species, Shannon, Simpson, and Pielou evenness indices were all notably lower (*p* < 0.05). Furthermore, VTM mining has induced alterations in the community structure of endophytic bacteria within the root systems of *E. indica* and *Carex*, as opposed to the non-VTM mining area. Due to VTM mining, the abundance of *Nocardioides* in both *E. indica* and *Carex* notably reduced (*p* < 0.05). Interestingly, the abundance of *Pseudomonas* in the roots of both plants significantly increased (*p* < 0.05), indicating that this bacterium has good environmental adaptability. Through PICRUSt2 prediction, our study reveals that the endophytic bacteria in the root systems of *E. indica* and *Carex* in the VTM mining area responded to the environmental stress caused by mining activities through a series of methods. These include the enhancement of β-Lactam resistance (ko00312), flavonoid biosynthesis (ko00941), polymyxin resistance (PWY0-1338), and nicotinate degradation I (PWY-722). Conversely, no substantive alterations were observed in the diversity, community composition, and metabolic functionality of endophytic bacteria within the root tissue of *D. viscosa* following VTM mining activities. This shows that *D. viscosa* has good environmental adaptability. This paper, for the first time, studied the effect of VTM mining activities on endophytic bacteria in nearby plant roots. It emphasizes the ecological impact of VTM mining activities on endophytic bacteria in plant roots and provides a basis for soil ecological remediation in VTM mining areas.

## Data Availability

Data are contained within the article.
